# The relationship between mTOR signalling pathway and recombinant antibody productivity in CHO cell lines

**DOI:** 10.1186/1472-6750-14-15

**Published:** 2014-02-17

**Authors:** Raihana Edros, Susan McDonnell, Mohamed Al-Rubeai

**Affiliations:** 1 School of Chemical and Bioprocess Engineering and Conway Institute of Biomolecular and Biomedical Research, University College Dublin, Dublin, Ireland

**Keywords:** CHO, mTOR, Monoclonal antibody, Phosphotidylinositol 3-kinase, Cell engineering

## Abstract

**Background:**

High recombinant protein productivity in mammalian cell lines is often associated with phenotypic changes in protein content, energy metabolism, and cell growth, but the key determinants that regulate productivity are still not clearly understood. The mammalian target of rapamycin (mTOR) signalling pathway has emerged as a central regulator for many cellular processes including cell growth, apoptosis, metabolism, and protein synthesis. This role of this pathway changes in response to diverse environmental cues and allows the upstream proteins that respond directly to extracellular signals (such as nutrient availability, energy status, and physical stresses) to communicate with downstream effectors which, in turn, regulate various essential cellular processes.

**Results:**

In this study, we have performed a transcriptomic analysis using a pathway-focused polymerase chain reaction (PCR) array to compare the expression of 84 target genes related to the mTOR signalling in two recombinant CHO cell lines with a 17.4-fold difference in specific monoclonal antibody productivity (*q*_*p*_). Eight differentially expressed genes that exhibited more than a 1.5-fold change were identified. *Pik3cd* (encoding the Class 1A catalytic subunit of phosphatidylinositol 3-kinase [PI3K]) was the most differentially expressed gene having a 71.3-fold higher level of expression in the high producer cell line than in the low producer. The difference in the gene’s transcription levels was confirmed at the protein level by examining expression of p110δ.

**Conclusion:**

Expression of p110δ correlated with specific productivity (*q*_*p*_) across six different CHO cell lines, with a range of expression levels from 3 to 51 pg/cell/day, suggesting that p110δ may be a key factor in regulating productivity in recombinant cell lines.

## Background

The production of biopharmaceutical products is one of the fastest growing areas in the pharmaceutical industry with sales of $99 billion in 2010 [1,2]. Many of these biopharmaceutical products are used to treat critical diseases, like hepatitis, cancer, heart disease, hemophilia, rheumatoid arthritis, and diabetes. Monoclonal antibodies (mAbs) currently constitute more than 30% of total biopharmaceuticals and the portfolio of products is expected to grow between 7-15% per annum in the coming years. Whilst the future does looks bright for this sector, the emergence of biosimilar competition is ensuring that manufacturers of these products are continuously seeking methods of increasing productivity of cell culture processes to maximise yields.

Numerous attempts have been made to generate cell lines that can produce large amounts of recombinant products. Among the cell lines available for mAb production are myeloma, hybridoma, and Chinese hamster ovary (CHO) cell lines [3,4]. The use of CHO cells in large-scale production is common [3,5-7] because of their ability to express high recombinant protein levels [8,9], grow to high cell densities [10-13], and to grow in serum-free suspension culture [6,14-16]. CHO cells are also suitable for use with expression systems, such as dihydrofolate reductase (DHFR) and glutamine synthetase (GS) [17-19].

High productivity is usually accompanied by slower growth rates [20-25], and generating high-producing cell lines with high growth rates should be advantageous to further improve mAb production. However, maintaining cell-specific productivity at high growth rates is challenging because both processes require high energy. Glutamine and glucose are often consumed in the synthesis of precursors for recombinant proteins rather than in cell proliferation [26], which eventually leads to growth repression.

The correlation between cell volume (size) and productivity has been clearly demonstrated [27] and is reflected in the phenotypic changes in protein content and cell biomass [13,28,29]. This increase in protein content may lead to an increase in cell size [25,29,30]. The integration of both protein synthesis and cell proliferation in response to diverse environmental cues has been reported to be regulated by a signalling pathway known as the mammalian target of rapamycin (mTOR) signalling pathway [31,32].

**Figure 1:**
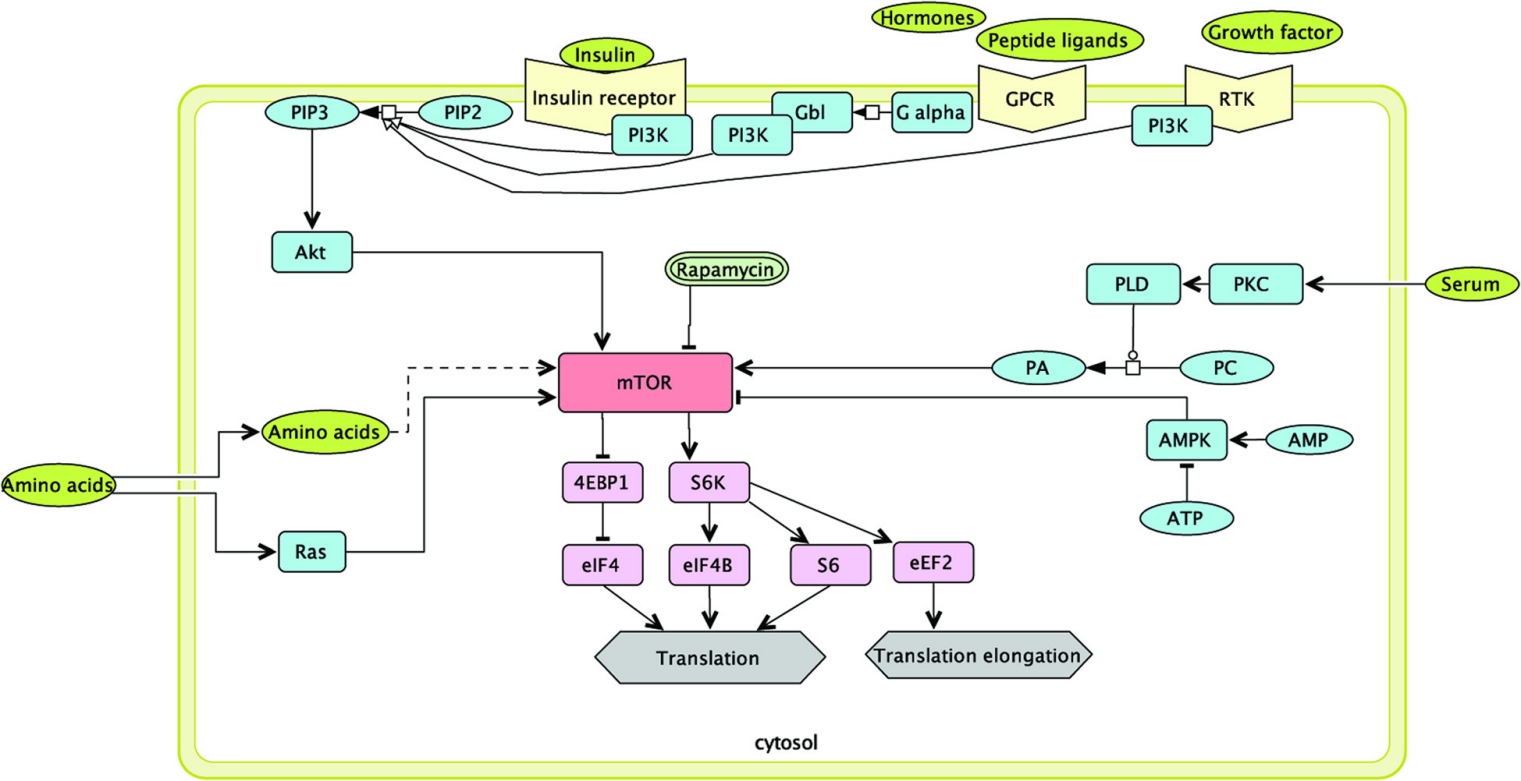
**The mTOR pathway signaling pathway.** This pathway involves communication between the upstream proteins that respond directly to such extracellular signals as nutrient availability, energy status, and physical stresses.

As shown in Figure 1, upon receiving signals from growth factors and hormones, mTOR is activated by Akt/P13K signalling [33,34]. P13K catalyses the formation of phosphoinositides-3-phosphate (PIP3) from phosphoinositides-2-phosphate (PIP2), which then activates a protein kinase known as Akt [35]. The activated mTOR initiates protein synthesis via two principal pathways; firstly through the inhibition of eukaryotic initiation factor 4E binding protein (4EBP1) and secondly through the activation of ribosomal protein S6 kinase (S6K) [36,37]. These downstream effectors activate protein synthesis by activating translation initiation and elonga tion factors, including eIF4E, eIF4B, S6, and eEF2 [32,38].

In addition to growth factors and hormones, mTOR also receives signals from phosphatidic acid (PA) [39] and amino acids [38,40,41]. In the presence of serum, protein kinase C (PKC) is activated, and it sequentially activates phospholipase D (PLD) which is responsible for converting PA to phosphatidylcholine (PC) [39]. In contrast, the activation of mTOR by amino acids is unclear [32]. It was reported to be regulated by the Ras protein [41] and/or intracellular amino acids [38,42]. Moreover, adenosine monophosphate kinase (AMPK) can also precisely modulate mTOR activity based on the adenosine monophosphate (AMP) to adenosine triphosphate (ATP) ratio [43]. In the presence of high intracellular ATP levels, AMPK can be deactivated, leading to an increase in protein synthesis [44,45].

A recent study highlighted the role of mTOR in improving viability, cell growth, antibody production, and robustness of the CHO cell line [46]. However, the mechanism underlying these improvements has not yet been elucidated. We believe that using a transcriptomic approach, such as the pathway-focused polymerase chain reaction (PCR) array, which has proven to be useful in a similar study [47], we can elucidate the connections between mTOR, cell growth, and productivity. Thus, this study initially employed two recombinant CHO cell lines with a 17.4-fold difference in specific productivity to study the differential expressions of genes related to the mTOR signaling pathway. High productivity has been associated partly with enhanced translation mechanisms [48]. We therefore expected to see upregulation of genes encoding mTOR upstream regulators such as, Akt [49,50], PI3K [51,52], PLD [39,53], and Ras-related GTP binding proteins (RagC) [41,54,55], as well as downstream effectors involved in translation mechanisms such as S6 and S6K [56-59]. Since high gene expression does not necessarily mean that the corresponding protein is also highly upregulated in the cell, we decided to perform Western blot analysis to confirm that the selected potential marker p110δ was expressed in increasing amount in a panel of 6 CHO cell lines with varying levels of productivity.

## Methods

### Cell lines and maintenance

This study employed a panel of six GS-CHO cell lines (referred to as CL38, CL47, CL76, CL150, CL160 and CL164) producing cB72.3 IgG4 monoclonal antibody with varying levels of productivity. The cell lines were kindly provided by Lonza Biologics (Slough, UK). The cell lines were generated by transfection of the suspension-variant derivatives CHO-K1 (CHOK1SV) with the glutamine synthetase (GS) expression vector pcB72.3 containing light chain (LC) and heavy chain (HC) cDNA, each driven by the hCMV-MIE promoter [60,61]. Cells were maintained in CD-CHO medium supplemented with 25 μM MSX. Using three biological replicates, our experiments were conducted in 125 ml shake flasks (SCHOTT Inc., Elmsford, USA) at 37°C and 140 rpm agitation.

The six cell lines were numbered in descending order according to their specific productivity, with [1] referring to the most productive cell line and [6] referring to the least productive cell line. These cell lines were thus referred to as CL47[1], CL76[2], CL150[3], CL164[4], CL38[5] and CL160[6] throughout this study . Based on specific productivity, CL47[1] was categorised as the high producer; CL76[2], CL150[3], CL164 [4], and CL38[5], as medium producers; and CL160[6], as the low producer.

### Flow cytometric analysis of cell number and viability

Cell suspensions (490 μl) were removed from flasks and placed in a flow cytometry tube and 10 μl propidium iodide (PI) solution added from the stock solution (0.5 μg/ml). Samples were mixed by gentle shaking and analysed immediately for cell numbers and viability, using Cell Lab Quanta SC flow cytometry (Beckman Coulter Inc., CA, USA) equipped with an argon laser (488 nm). Red fluorescence (PI) was collected using a 635 nm band pass filter. Analysis was undertaken by loading an appropriate protocol for the acquired parameters: electronic volume (EV), log side scatter (SS), and PI integral; 10 000 observations were collected for analysis. The evaluation of cell number was achieved by gating areas in the EV versus log SS dot plot in which living cells and dead cells appear.

We used the following equation to calculate the specific growth rate, *μ*:

$$\mu =\frac{\left(\ln X_{V_{1}}-\ln X_{V_{0}}\right)}{t_{1}-t_{0}}$$

Where $X_{V_{1}}$ and $X_{V_{0}}$ symbolise the viable cell density at time points *t*_*1*_ and *t*_*0*_, respectively.

### Determination of antibody concentration

Enzyme-linked immunosorbent assay (ELISA) was used to determine the concentration of mAb secreted by the cell lines, as described previously [30]. The antibody was sandwiched between monoclonal anti-human IgG Fc (Sigma-Aldrich, St. Louis, USA) and anti-human kappa light chain horseradish peroxidase (HRP) conjugates (Sigma-Aldrich). The concentration of antibody in the samples was determined using o-phenylenediamine (OPD; Sigma-Aldrich) as a substrate, and the specific productivity, *q*_*p*_, was calculated using the following equation:

$$q_{p}=\frac{{\left[\mathrm{MAb}\right]}_{1}-{\left[\mathrm{MAb}\right]}_{2}}{\mathrm{CVCT}}$$

where [MAb] represents the antibody concentration at a particular time, while cumulative viable cell time (CVCT) is the sum of the individual areas given by

$$\mathrm{CVCT}=\sum \left(\left(\frac{x_{1}+x_{0}}{2}\right)\times \left(t_{1}-t_{0}\right)\right)$$

Where *x*_*0*_ represents the cell number at the particular time *t*_*0*_ and *x*_*1*_ represents the cell number at the elapsed time *t*_*1*_.

### Quantitative real-time PCR (qRT-PCR) analysis

3 × 10^6^ cells were removed from the flask at the mid-exponential phase (day three of the batch culture), centrifuged, and washed twice by PBS. The sample was treated with RNA*later* and stored at −80°C until analysis, at which point it was centrifuged to remove the RNA*later* stabilization reagent. RNA isolation was carried out using the RNeasy Mini Kit (QIAGEN, Valencia, CA, USA) according to the manufacturer’s instructions. The concentration of RNA was determined using a NanoDrop ND-1000 UV–vis Spectrophotometer (Nanodrop Technologies, Wilmington, DE, USA), and the integrity of RNA was checked using an Agilent Bioanalyzer (Santa Clara, CA, USA).

The expression levels of mTOR-related genes were quantified using a mouse-mTOR-pathway-focused qRT-PCR array from SA Biosciences (Frederick, Maryland, USA). The DNA elimination treatment was carried out, and complementary DNA (cDNA) was synthesized from the RNA samples, using the RT^2^ First Strand Kit (SA Biosciences) according to the manufacturer’s instructions. The cDNA samples were mixed with RT^2^ SYBR Green/ROX qRT-PCR Master Mix reagents (SA Biosciences) according to the manufacturer’s instructions, and the qRT-PCR was performed on these samples using ABI Prism 7500 FAST sequence detection system (Applied Biosystems, Carlsbad, CA, USA). The C_t_ values obtained from the qRT-PCR analysis were normalised to five housekeeping genes (beta glucuronidase [*Gusb*], hypoxanthine quinine phosphoribosyl transferase 1 [*Hprt1*], heat shock protein 90 alpha [*Hsp90ab1*], glyceraldehyde-3-phosphate dehydrogenase [*Gapdh*], and beta actin [*Actb*]). The normalization was calculated by using the *2*^*-∆∆CT*^ method, as previously described [62].

### Total protein content

The total protein content was determined using the QuantiPro BCA Assay Kit (Sigma-Aldrich). 3 × 10^6^ cells were removed from the flask, centrifuged and then washed once with sterile-filtered PBS. The cells were then centrifuged at 500 g for five minutes and supernatant removed. A mixture of CelLytic M Cell Lysis Reagent (Sigma-Aldrich) and a protease inhibitor cocktail (Sigma-Aldrich) was added to disrupt the cells. The samples were mixed by vortexing for one minute, centrifuged at 15 000 g for 15 minutes at 4°C. The supernatant was transferred to a new microcentrifuge tube and stored at −20°C until analysis.

### Western blot analysis

For western blot analysis, 20 μg of total protein was mixed in 2× Laemmli buffer (Sigma-Aldrich) and denatured at 95°C. The samples were then subjected to electrophoresis on an 8% polyacrylamide gel (Thermo Scientific, Waltham, MA, USA) using a Mini Protean 3 electrophoresis system (Bio-Rad Laboratories, Waltham, MA, USA) for 90 minutes. The proteins were transferred electrophoretically to a PVDF membrane (Millipore Corporation, Cheshire, NH, USA) overnight. The membrane was blocked in non-fat milk powder in PBST followed by an overnight incubation with an anti-mouse p110δ PI3K antibody (Santa Cruz Biotechnology, Santa Crus, CA, USA). The membrane was probed with a secondary antibody solution containing anti-goat IgG peroxidase conjugates prior to detection using a SuperSignal West Pico kit (Thermo Scientific) according to the manufacturer’s instructions. Upon reaction, the membrane was exposed, developed, and fixed on a film. The band intensity was measured by the AlphaDigiDoc RT2 gel documentation system software (Alpha Innotech Corporation, CA, USA).

## Results

### Comparing the growth and productivity of the high and low producers

**Figure 2:**
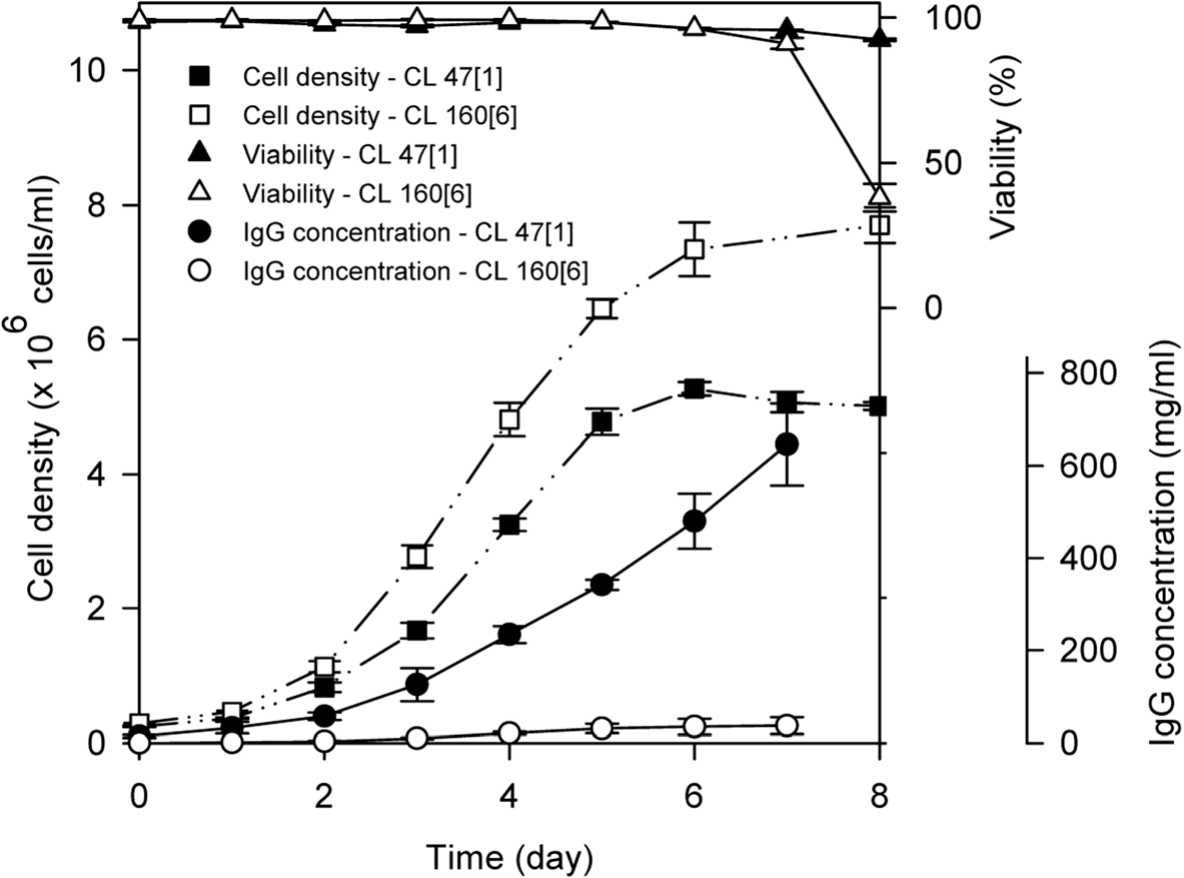
**Viable cell density, viability, and antibody concentration profiles of high producer (CL47[1]) and low producer (CL160[6]) cells lines during batch culture.** Error bars symbolize the standard deviation as calculated between three biological replicates.

The C47 [1] and CL160 [6] lines, which expressed the recombinant chimeric IgG4 mAb, were grown in batch cultures in three biological replicates. The cell numbers and antibody concentrations, determined by flow cytometric analysis and ELISA, respectively, were determined routinely throughout the culture duration until the cells reached the death phase. The cell growth rates and cumulative viable cell time (CVCT) were determined throughout the exponential phase, from day one to day five of batch culture as shown in Table 1. The viable cell density, viability, and antibody concentration profiles are shown in Figure 2. Following a normal growth curve, CL160[6] outperformed CL47[1] in terms of growth, as demonstrated by the calculated CVCT and growth rate (*μ*) of 329.66 ± 17.99 (10^9^ cell.h/L) and 0.67 ± 0.01 (day^-1^) in CL160[6], compared to 244.48 ± 4.58 (10^9^ cell.h/L) and 0.63 ± 0.01 (day^-1^) in CL47[1], respectively. However, CL47 [1] sustained high viability towards the decline phase (day 8), while the viability of CL160[6], on the contrary, started to decrease sharply on day seven. In CL 47[1], the specific productivity and total antibody concentration, as indicated in Table 1, were 17.4- and 11-fold higher than CL160[6], respectively.

**Table 1:** The culture characteristics and cell productivity of high producer (CL47[1]) and low producer (160[6]) cell lines during batch culture

	**47[1]**	**160[6]**
CVCT (10^9^ cell.hr/L)	244.48 ± 4.58	329.66 ± 17.99
Antibody concentration (μg/ml)	575.24 ± 137.55	52.08 ± 0.77
Growth rate (day^-1^)	0.63 ± 0.01	0.67 ± 0.01
*q*_*p*_ (pg/cell/day;pcd)	50.54 ± 3.18	2.91 ± 1.45

The symbol “±” represents the standard deviation calculated between the three biological replicates.

### Differential mTOR-related gene expression between high and low producers

**Figure 3:**
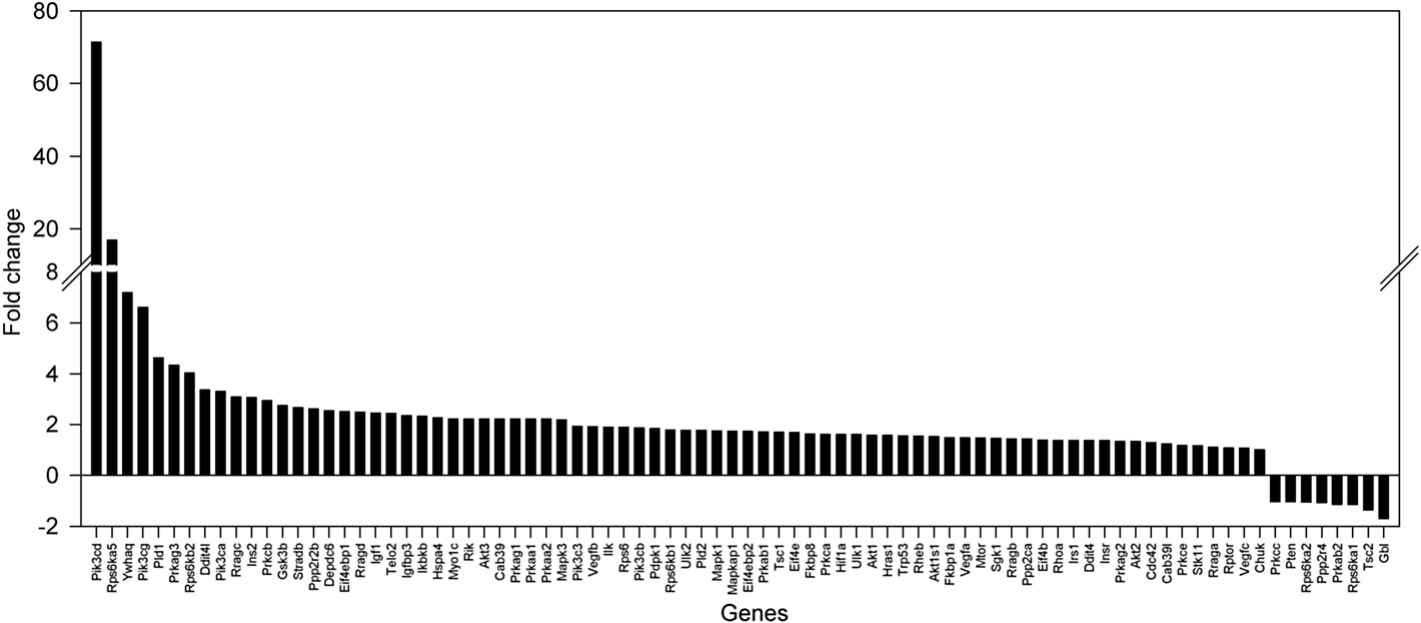
**The fold changes of mTOR-related gene expressions of GS-CHO cell lines at the mid-exponential phase of batch culture.** Values were calculated based on a relative quantification made between high producer (CL47[1]) and low producer (160[6];control) lines by using the 2^-∆∆CT^ method.

**Figure 4:**
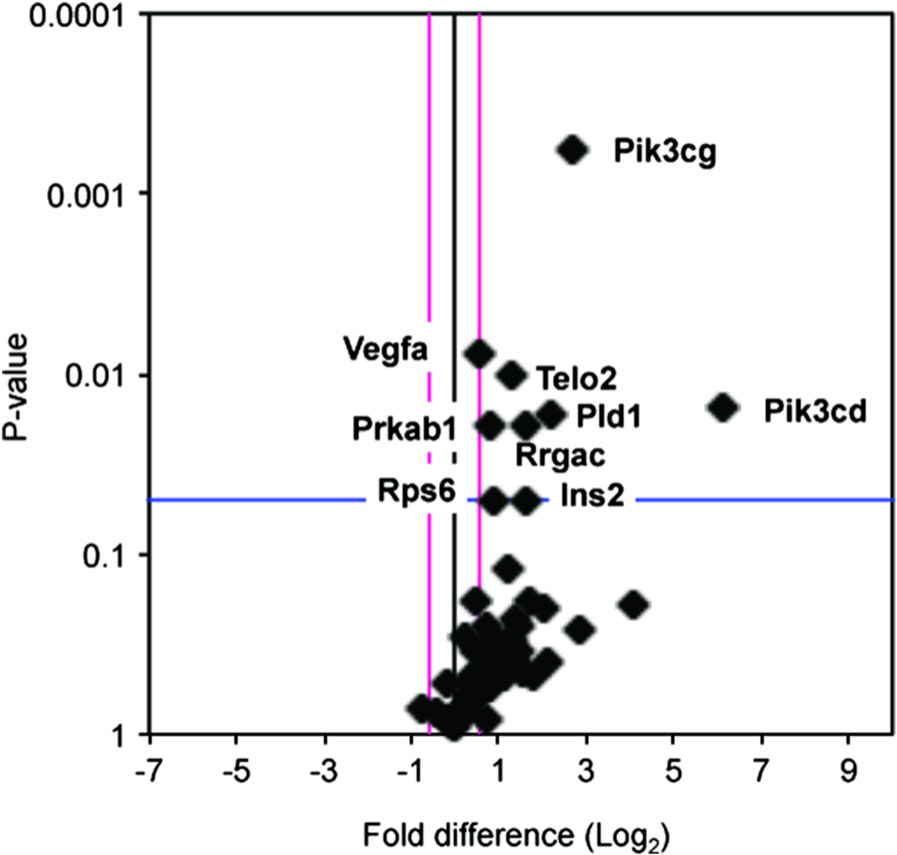
**The log**_**2 **_**fold changes in gene expression between high producer (CL47[1]) and low producer (160[6]) lines against t-test P-values.** Vertical line represents the threshold of fold changes (i.e., 1.5); horizontal line represents the significant difference of t-test P-values (i.e., 0.05). Significantly expressed genes (P-values < 0.05) are tabulated in Table 2.

We hypothesized that the differences in CL47 [1] and CL160[6] productivity levels are a consequence of the diverse mechanisms of cellular functions that are controlled by mTOR. Using a commercially available pathway-focused PCR array, the expression of 84 genes related to the mTOR signalling pathway were screened in the template cDNA which was prepared from the samples collected at the mid-exponential phase (day three of the batch culture). Figure 3 demonstrates the relative expression of the mTOR-related genes exhibited by the high producer (CL47[1], treated) in comparison to the low producer (CL160[6], control). Student’s t-tests were conducted to determine the significance of the gene expression changes. Out of the 84 mTOR-related genes in this array, eight genes expressed at least a 1.5-fold difference (*Pik3cd, Pik3cg, Pld1, Rragc, Ins2, Telo2, Rps6* and *Prkab1*), with a corresponding statistical significance (P-value < 0.05) between the high and low producers (Figure 4). These genes were all upregulated.

### Expression of genes in mTOR signalling pathway

The list of mTOR-related genes with significantly altered expression in CL47[1] relative to CL160[6] is presented in Table 2. Three differentially expressed genes involved in insulin signalling were upregulated. The most upregulated gene, *pik3cd*at 71.3-fold (P-value < 0.05), encodes the catalytic subunit of the delta polypeptide p110δ. The gene encoding a p110δ sibling, the gamma polypeptide (p110γ) subunit, was also significantly expressed but to a lesser extent (6.6-fold; P-value < 0.05). The p110δ and p110γ subunits are classified as Class 1A and 1B, respectively and both receive signals from insulin and various types of growth factors [51,63,64]. Another gene related to insulin signalling that was also significantly expressed was insulin 2 (*Ins2*), with a 3.06-fold change (P-value < 0.05).

**Table 2:** The mTOR-related genes with at least a 1.5-fold change in expression level (P-values < 0.05)

**Gene symbol**	**Description**	**Fold change**	**P-value**
*Pik3cd*	Phosphatidylinositol 3-kinase catalytic delta polypeptide	(+) 71.33	0.02
*Pik3cg*	Phosphoinositide-3-kinase, catalytic, gamma polypeptide	(+) 6.59	<0.001
*Pld1*	Phospholipase D1	(+) 4.63	0.02
*Rragc*	Ras-related GTP binding C	(+) 3.08	0.05
*Ins2*	Insulin II	(+) 3.06	0.02
*Telo2*	TEL2, telomere maintenance 2, homolog (S. cerevisiae)	(+) 2.44	0.01
*Rps6*	Ribosomal protein S6	(+) 1.89	0.05
*Prkab1*	Protein kinase, AMP-activated, beta 1 non-catalytic subunit	(+) 1.71	0.02

Ras-related GTP-binding protein C (*Rragc*) and phospholipase D1 (*Pld1*) were also expressed significantly, with fold changes of 3.08 and 4.63, respectively (P-value < 0.05). Unlike RagC, which has a response specific to amino acid availability [41,54,55], PLD1 is activated by mitogens such as serum [39]. Moreover, the expression of the regulatory gamma subunit of AMPK gene (*Prkag3*) was also observed to be significantly differentially expressed (1.71-fold, P-value < 0.05) though the expression was observed to be minimal compared to other significant differential mTOR-related genes. The activity of AMPK, precisely controlled by the ratio of AMP to adenosine triphosphate (ATP), reflects the cell energy status [43].

Among the genes that encode for downstream effectors of mTOR, only ribosomal protein S6 (*Rps6*) was significantly differentially expressed (1.89-fold, P-value < 0.05) in CL47[1] relative to the CL160[6]. The interactions among the upstream regulators, mTOR, and the downstream effectors are indicated in Figure 1. Although mTOR was not significantly expressed in this study, a gene that could be linked to its presence, known as *Telo2*, was upregulated by 2.44-fold (P-value < 0.05). This gene encodes telomere maintenance 2 (TEL2), which is responsible for mTOR stabilisation and maturation [65,66]. However, the mechanism by which TEL2 stabilizes mTOR remains poorly understood [65,66].

### The relationship among p110δ expression and specific productivity, cell growth, and cell size

**Figure 5:**
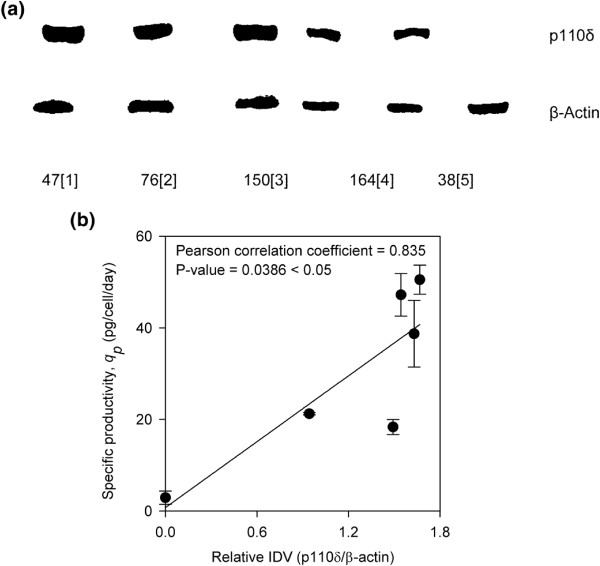
**Comparison of p110σ subunit expressions between (a) six GS-CHO cell lines with different productivity levels at the mid-exponential phase of batch culture and (b) the correlation between p110σ expression and *q***_***p***_**.** Error bars symbolize the standard deviation as calculated between three biological replicates.

To investigate whether *Pik3cd* mRNA levels in the high and low producers are predictive of p110δ expression, western blot analysis was performed on samples harvested from the mid-exponential phase (day three) of the batch cultures. Figure 5a shows the expression of the p110δ subunit in GS-CHO cell lines with different *q*_*p*_ levels ranging from 18 to 40 pg/cell/day. (CL47[1] = 50.54 pcd; CL76[2] = 47.23 pcd; CL150[3] = 38.73 pcd; CL164[4] = 21.23 pcd; CL38[5] = 18.34 pcd and CL160[6] = 2.91 pcd). The molecular characteristics of these cell lines have been extensively studied previously [67]. The integrated density values (IDV) corresponding to the band intensities were measured. Pearson correlation coefficient analysis was performed to determine the relationship between p110δ subunit expression and the *q*_*p*_ levels. The analysis revealed that the expression of the p110δ subunit correlated with the overall *q*_*p*_ (ρ = 0.83, P-value < 0.05; Figure 5b). This demonstrates the possibility that the p110δ subunit could be a potential marker describing *q*_*p*_. Previous studies have demonstrated that PI3K overexpression could influence cell growth and proliferation [68-70]. Thus, we attempted to correlate expression of the p110δ subunit with cell size and CVCT. A Pearson correlation coefficient analysis indicated that neither cell size nor CVCT (data not shown) correlated with the expression of the p110δ subunit (P-value > 0.05).

## Discussion

Since Dreesen and Fussenegger [46] recently showed that overexpression of mTOR in a CHO cell line significantly improved recombinant antibody production, we decided to investigate expression of members of the mTOR signalling in a panel of CHO cell lines with different levels of productivity. In order to address this question we used the mTOR signalling PCR array for mouse due to the unavailability of the hamster array at that time. Given that the mTOR signalling pathway is conserved from yeast to mammals [71], we used mouse sequences instead of hamster to quantify the levels of mTOR-related gene expressions in this study. Previous transcriptomic studies which relied on cross-hybridization to mouse DNA microarrays have showed considerable success [72] and in general, results from alignment to both mouse and rat reference genomes suggest that CHO genomic sequences are generally more similar to mouse genomic sequences [73]. Of the 84 mTOR-related genes present on the array only eight genes including, Pik3cd, Pik3cg, Ins2, Pld1, Rragc, Prkag3, Rps6 and Telo2 were identified as significantly (p < 0.05) upregulated in CL47[1] in comparison to CL160[6]. This panel of candidate genes could potentially determine high specific productivity in CHO cell lines. Two of the genes that were highly expressed, *Pik3cd* and *Pik3cg,* encode for p110δ and p110γ polypeptides, respectively and are also present on the SA Biosciences hamster mTOR signalling PCR array (PAJJ-098Z). These polypeptides differ at the regulatory subunit structure that is responsible for mediating p110δ and p110γ recruitment to the receptors of interest. The presence of p110δ as a regulatory subunit facilitates the binding of p110γ to the G protein beta subunit-like (Gβl) in response to a stimulated G-protein couple receptor (GPCR). The recruitment of the p110δ subunit to the activated receptor tyrosine kinase (RTK) is, however, mediated by a different regulatory subunit, p85, in response to various extracellular growth factors and insulin signals [35,74,75]. Hence, the different receptors as targets imply that upregulation of *pik3cd* gene could be independent of *pik3cg* (and vice versa), even though these polypeptides share a common role in catalysing phosphorylation of the inositol ring at the D3 position of their downstream effectors, the phosphoinositides.

Although the effects of p110δ in recombinant protein production have yet to be clarified, its associations with growth are better understood. The effects of p110δ overexpression have been correlated to cell growth and cell size in *Drosophila*, where expansion of the wing blade was due to an increase in cell number and cell size. A reverse effect on cell growth was observed in p110δ-deficient cells [68]. The real cause, which affects the lack of correlation between the CVCT and cell size with p110δ expression, is not clear. The correlation could be weakened by the presence of the medium producer cell line in which other signalling pathways could have compensated for the lack of effects of p110δ expression on cell growth. However, this expression effect is more profound when only two extremes (high and low producers) were considered. CL160 [6] has a smaller cell size (data not shown) than CL47[1], and this could partly result from the defective p110δ expression in CL160[6] [51,68]. There has been some studies published which examined the use of genetic control of the cell cycle to increase *q*_*p*_. However, these studies cannot confirm that the production of recombinant protein is related to any specific phase of the cell cycle. However, the increase in *q*_*p*_ can be related to cell size rather than the cell cycle phase [17]. This has been shown previously by Bi et al. [30] in a cytostatic system utilising inducible expression of p21^CIP1^ to arrest CHO cells where a fourfold increase of *q*_*p*_ was obtained concomitant to a fourfold increase in cell volume. Regardless of cell cycle phase, the cell size could also be controlled by p110δ expression through protein synthesis, a mechanism that is important for cell growth and proliferation. As cells have to retain a constant cell size during proliferation, a tight coordination between cell growth and cell cycle progression is essential for maintaining cell size and these processes are controlled by p110δ expression as the upstream regulator of mTOR signalling pathway [33,76,77].

While the regulation of p110δ activity is related to cell growth, the activity of p110γ involves the regulation of cell survival [78-80]. An increased resistance to cell death was also observed as a result of the simultaneous inhibition of p53 and activation of nuclear factor κB (NF-κB) signalling pathways by Akt phosphorylation following the overexpression of the p110γ subunit in human cell lines [80].

In addition, *Prkag3, Pld1,* and *Rragc* genes were also significantly expressed in CL47 [1], and these could be related to high specific productivity. These genes encode AMPK, PLD, and Ras-related GTP-binding protein C, which represent upstream regulators of mTOR. The altered expression of these genes may implicate the expression of the *Rsp6* gene, which encodes the S6 protein. The S6 protein regulates the translation of ribosomal protein, elongation factor, and polyA-binding protein, that could lead to ribosome biogenesis [81-83]. This suggests that the improved specific productivity in CL47 [1] could be due to the altered expression of the *Rsp6* gene. Our results were supported by a study conducted by Bi et al. [30]. A significant increase in mAb titre was shown to correlate with higher S6 protein expression in an isopropyl-β-d-thiogalactoside (IPTG)-induced p21^cip^-arrested CHO cell line [30].

We also found altered expression of the *Ins2* gene in the CL47[1] cell line; this gene is not commonly expressed in non-pancreatic β cells. It is known that all cells contain an insulin gene, but its expression can vary in different cell types. The expression of *Ins2* could be controlled at the transcriptional level. It was shown previously by Kuroda et al. [84] that the *Ins2* gene was fully methylated and becomes demethylated as the cells differentiate into insulin-expressing cells *in vitro*[84]. If CHO cells could produce insulin, this would make the cells less dependent on exogenous insulin, which is certainly an advantageous trait in recombinant protein production.

## Conclusion

This study provides a better understanding of the role of the mTOR signalling pathway in GS-CHO cell lines and identifies the importance of this pathway in recombinant protein production. We also highlight the presence of the p110δ subunit of PI3K as a potential key regulator in productivity that may represent a future target for cell-engineering strategies for enhancing productivity. Cell-engineering approaches such as overexpression and/or gene silencing could be used to verify the roles of p110δ and p110γ in cellular functions that may be involved in recombinant protein production.

## Competing interests

The authors declare no competing interests.

## Authors’ contribution

RE carried out the molecular genetic and cell culture studies, and participated in the sequence alignment. All authors wrote, edited and approved the manuscript.
